# Capacitance Effects of a Hydrophobic-Coated Ion Gel Dielectric on AC Electrowetting

**DOI:** 10.3390/mi12030320

**Published:** 2021-03-18

**Authors:** Taewoo Lee, Sung-Yong Park

**Affiliations:** 1 Department of Mechanical Engineering, National University of Singapore, 9 Engineering Drive 1, Singapore 117576, Singapore; taeulee@gmail.com; 2 Department of Mechanical Engineering, San Diego State University, 5500 Campanile Drive, San Diego, CA 92182, USA

**Keywords:** AC electrowetting, ion gel, contact angle modulation, droplet oscillation, high capacitance

## Abstract

We present experimental studies of alternating current (AC) electrowetting dominantly influenced by several unique characteristics of an ion gel dielectric in its capacitance. At a high-frequency region above 1 kHz, the droplet undergoes the contact angle modification. Due to its high-capacitance characteristic, the ion gel allows the contact angle change as large as Δ*θ* = 26.4°, more than 2-fold improvement, compared to conventional dielectrics when *f* = 1 kHz. At the frequency range from 1 to 15 kHz, the capacitive response of the gel layer dominates and results in a nominal variation in the angle change as *θ* ≈ 90.9°. Above 15 kHz, such a capacitive response of the gel layer sharply decreases and leads to the drastic increase in the contact angle. At a low-frequency region below a few hundred Hz, the droplet’s oscillation relying on the AC frequency applied was mainly observed and oscillation performance was maximized at corresponding resonance frequencies. With the high-capacitance feature, the ion gel significantly enlarges the oscillation performance by 73.8% at the 1st resonance mode. The study herein on the ion gel dielectric will help for various AC electrowetting applications with the benefits of mixing enhancement, large contact angle modification, and frequency-independent control.

## 1. Introduction

Using the surface tension force dominance over body forces in micro/meso scales, electrowetting has been explored as an effective means for small-scale liquid handling without complex mechanical components such as pumps, tubes, and microchannels [1,2,3]. When an electric potential is applied between a droplet and an electrode, the electric charges are re-distributed and modify the surface tension at the liquid-solid interface where the like-charge repulsion reduces the work by expanding the surface area [4,5]. The resulting contact angle (*θ*) of a liquid droplet can be mathematically estimated with the applied electric potential (*V*) by using the popular Young−Lippmann equation [6]:

(1) $$\cos\theta =\cos\theta_{0}+\frac{1}{2\gamma }cV^{2}$$

where *θ*_0_ is an initial contact angle with zero potential application, *γ* is the surface tension between two immiscible fluids, and *c* is the specific capacitance of the dielectric layer which is proportional to its relative permittivity (*ε*_r_) and the inverse of the layer thickness (*t*). Using the electrowetting mechanism, numerous applications have been demonstrated, including lab-on-a-chip [7,8,9], tunable optics [10,11,12], energy harvesting [13,14,15], and on-chip cooling [16,17].

According to the Young-Lippmann Equation (1), electrowetting performance (i.e., contact angle modulation) is determined by the capacitance of a dielectric layer used. The use of high-capacitance dielectrics is very beneficial to induce large contact angle modification at a given potential input by allowing high electrostatic energy (*cV*^2^/2) stored across a capacitor of a dielectric layer. To meet this requirement, our group recently presented an ion gel as a low-cost, spin-coatable, high-capacitance dielectric [18]. An ion gel is a solid thin-film form of an ionic liquid, being fulfilled by combining it with a structuring copolymer [19,20]. Since the ionic liquid is an essential constituent of the ion gel, it possesses similar electrical properties to the ionic liquid. Under an electric field applied, free counter-ions inside the ionic liquid compactly accumulate at the liquid-solid interface where the nanometer-thick electric double layer capacitor (EDLC) is formed [21]. This ultra-thin EDLC provides a large specific capacitance (*c* ≈ 10~40 μF/cm^2^), which provides 2 to 3 orders of magnitude higher than that of conventional dielectrics such as silicon dioxide (SiO_2_) [19,22]. Using such a high-capacitance feature of the ion gel, our previous study has demonstrated significant improvement in electrowetting performance under an application of a direct current (DC) voltage [18].

For typical electrowetting studies [4,23,24], a liquid droplet is equivalently modeled as a resistor and a capacitor connected in parallel, while a dielectric layer underneath a droplet is equivalently assumed as a capacitor. Under a DC situation where an ohmic current dominates, the droplet behaves as a perfect conductor and the applied electric potential fully contributes to the contact angle reduction of the droplet. A resulting contact angle modification can be reasonably estimated by using the Young-Lippmann Equation (1). However, when an alternating current (AC) voltage applied, this general understanding is no longer valid because a displacement current dominates. An alternating electric input results in several interesting AC electrowetting phenomena such as droplet oscillation [25,26] and internal flow [27] that cannot be simply explained using the Young-Lippmann Equation (1). These AC electrowetting phenomena have been advantageously used for various AC electrowetting applications, including rapid mixing without the need of extra apparatus such as pumps or mixers, less contact angle hysteresis, and mitigation of contact angle saturation [28,29,30].

In this paper, we present AC electrowetting responses of a water droplet dominantly affected by several unique characteristics of an ion gel dielectric in its capacitance, such as high-capacitance magnitude, thickness independency, and frequency independency in a specific frequency region. To understand the effects of such features on AC electrowetting, experimental studies were conducted with a hydrophobic-coated ion gel layer at both high- and low-frequency AC regions. For comparative studies, the AC electrowetting performances on the gel layer were further compared to the ones on conventional dielectrics such as SiO_2_ fabricated by a sputtering process. The study herein on the ion gel dielectric will help for various AC electrowetting applications with the benefits of mixing enhancement, large contact angle modification, and frequency-independent control.

## 2. Fundamentals of an Ion Gel Dielectric for AC Electrowetting

Ionic liquids are generally known as room temperature molten organic salts [21,31,32]. Due to their favorable properties such as wide electrochemical window, high thermal stability, negligible vapor pressure, and large ionic capacitance, ionic liquids have been used for numerous applications of batteries, solar cells, and lubricants [33,34,35]. To further extend their practical applications, ionic liquids have been also fabricated in a solid thin-film form either by chemical or physical cross-linking with structuring polymers. This thin-film form of an ionic liquid is known as an ion gel. Hence, the ion gel retains similar electrochemical properties to the ionic liquid. In addition, the ultra-thin EDLC formed at an electrified surface allows a specific capacitance in a few orders of magnitude higher than that of conventional dielectrics.

**Figure 1:**
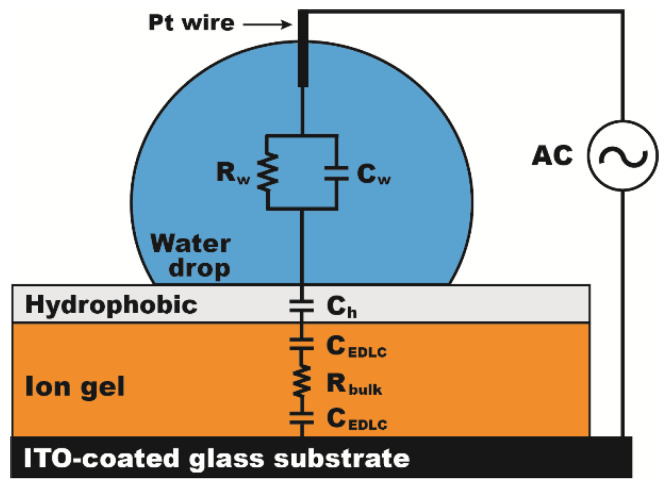
An experimental setup for alternating current (AC) electrowetting study with the ion gel. To investigate the gel layer effect on AC electrowetting, a 15 μL de-ionized water drop is placed on the ion gel layer covered by a thin hydrophobic layer. An equivalent circuit for the gel layer can be modelled as two capacitors (*c*_EDLC_) serially connected with a resistor (*R*_bulk_). A water drop is equivalently modelled as an impedance formed with a resistor (*R*_w_) and a capacitor (*c*_w_) connected in parallel, while a hydrophobic layer is modeled as a single capacitor (*c*_h_).

Based on previous studies [19,36,37], it was reported that the capacitance of the ion gel shows frequency-dependent behaviors very differently from that of conventional dielectrics. For AC electrowetting study, a typical dielectric layer is equivalently modeled as a single capacitor, while the ion gel dielectric layer can be modeled using an equivalent circuit consisting of capacitors and a resistor connected in series, as shown in Figure 1. The capacitor term (*c*_EDLC_) of the gel layer represents the compact EDLC formed at the electrified interface, while the resistor part (*R*_bulk_) is induced by a bulk electrolyte resistance emerging from the constituent ion liquid. This indicates that the capacitance of the gel layer is solely contributed by the thickness of the EDLC, not determined by the thickness of the gel layer itself. According to the Gouy-Chapman theory [21,31], the thickness of the EDLC is effectively estimated by using the Debye length (less than 10 nm for most ionic liquids), which can be used to determine the gel layer’s capacitance [31,38]. Furthermore, Lee et al. have experimentally discovered the frequency-dependent characteristics of the gel layer composed of a P(VDF-HFP) structuring copolymer (poly(vinylidene fluoride-co-hexafluoropropylene)) and an [EMIM][TFSI] ionic liquid (1-ethyl-3-methylimidazolium bis(trifluoromethylsulfonyl)imide) using impedance spectroscopy [19]. The impedance (Z) was measured in the form of Z = Z′ + *i*Z″, where *i* is an imaginary unit, and Z′ and Z″ constitute the real and imaginary parts of the impedance, respectively. Using this method, they found that the ion gel layer mainly behaves as a capacitor at the input AC frequency below around 15 kHz and its capacitance value was estimated *c* ≈ 10 μF/cm^2^ from the imaginary part (Z″) of the impedance. Above 15 kHz, the resistive response more dominates than the capacitive one and the value was calculated as *R* = 0.1~10 Ω cm^2^ from the real part (Z′) of the impedance. Considering the theoretical understanding described above, the ion gel dielectric possesses several unique characteristics in its capacitance under an AC input, such as high-capacitance magnitude, thickness independency, and frequency independency in a specific frequency region. In following sections, experimental studies will be discussed to understand how such ion gel’s features for the capacitance have an influence on AC electrowetting.

## 3. Experimental Setup

Figure 1 shows an experimental setup to investigate the effects of the ion gel dielectric on AC electrowetting performance. In this study, a P(VDF-HFP) structuring copolymer and an [EMIM][TFSI] ionic liquid were selected for the gel layer fabrication on an ITO substrate. A gel solution was first prepared by mixing a pellet of the copolymer in the ionic liquid at a ratio of 1:4 by weight. Acetone was added at a wt% ratio of 1:4 to further dilute the mixture. Then, the copolymer was fully dissolved in the acetone-mixed solution at 60 °C for 2 h. The mixture solution was then spin-coated on an ITO-coated glass substrate to obtain a thin-film gel layer. The layer thickness was controlled by varying a spin speed. The coated gel layer was cured at 75 °C for 4 h, after leaving it for 24 h at room temperature to ensure the vaporization of acetone. A Teflon solution (AF1600, Dupont, Wilmington, DE, USA) was subsequently spin-coated on top of the cured gel layer, followed by baking it at 75 °C for 16 h to deposit a 150 nm thick hydrophobic layer. A 15 μL deionized water droplet was placed on a hydrophobic surface and a platinum (Pt) wire was inserted into the droplet. A sinusoidal AC signal was applied between the Pt wire and the bottom ITO electrode. Its AC frequency (*f*) and root-mean-square voltage (*V*_RMS_) were variably controlled by a waveform generator (WW5061, Tabor Electronics, Nesher, Israel) and a high-voltage amplifier (9100A, Tabor Electronics, Nesher, Israel), respectively. AC electrowetting behaviors of a de-ionized water droplet were recorded using a high-speed camera (Fastcam Mini AX200, Photron, Tokyo, Japan) for further characterization. To highlight the effects of the gel layer on AC electrowetting, the experimental results with the gel layer were further compared to the ones on a hydrophobic-coated SiO_2_ layer deposited by sputtering at 150 W and 0.5 Å/s without a post-annealing process. All experiments were repeated four times for each experimental condition and the mean values were presented with the error bars for each case.

## 4. Results and Discussion

AC electrowetting phenomena are typically characterized in two input frequency regions, i.e., a high-frequency region above 1 kHz and another region at low frequencies below a few hundred Hz. Following sections will discuss about AC electrowetting responses of a water droplet influenced by the ion gel in both frequency ranges.

### 4.1. High-Frequency AC Electrowetting with the Ion Gel

**Figure 2:**
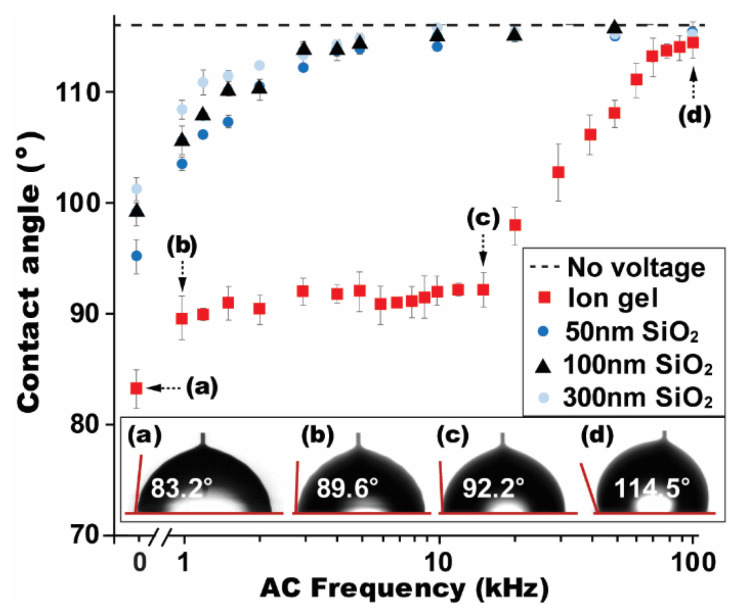
High-frequency AC electrowetting performance. Droplet’s contact angle was measured on various dielectric layers such as 8.3 μm ion gel and 50, 100 and 300 nm SiO_2_ covered with a 150 nm Teflon layer, while an applied AC frequency was varied from 1 to 100 kHz. Due to the high-capacitance dominance of the ion gel up to 15 kHz, the contact angle modulation is far superior, compared to SiO_2_. The AC electrowetting performance was further compared with DC cases where much lower contact angles are observed. All experiments were conducted at a fixed voltage of *V*_RMS_ = 42.4 V.

A high-frequency AC electrowetting study was firstly conducted on a SiO_2_ layer as a conventional dielectric. At the high-frequency region above 1 kHz where a displacement current becomes more dominant, a droplet acts in a capacitive manner due to limited dipole response time and thus the formation of an electric field across the droplet cannot be negligible [39,40]. As a result, the contact angle change is mainly observed under a high-frequency AC input. Figure 2 presents the contact angles experimentally measured while the applied AC frequency (*f*) was varied from 1 to 100 kHz. Without the voltage application, the droplet was placed on a hydrophobic surface with its initial contact angle of *θ*_0_ = 116.0° (indicated as a black dashed line in Figure 2). When *V*_RMS_ = 42.4 V and *f* = 1 kHz, it underwent the contact angle reduction to *θ* = 103.5° on a 50 nm SiO_2_ layer (i.e., angle change, Δ*θ* = *θ*_0_ − *θ* = 12.5°). With an increase in *f*, less contact angle change is observed. At *f* = 10 kHz, it returned almost close to an initial state of *θ*_0_. For thicker layers of SiO_2_, such a trend in the angle change becomes slower due to lower capacitance of the dielectric layers. For example, a 300 nm thick SiO_2_ layer allows the contact angle change as small as Δ*θ* = 7.5° at *f* = 1 kHz, although the same *V*_RMS_ = 42.4 V was applied. As further increasing *f*, the contact angle approached the initial state of *θ*_0_, similarly to other thicknesses of a SiO_2_ layer.

The experiment above was repeated for the gel layer and several interesting results were observed. When the same *V*_RMS_ = 42.4 V and *f* = 1 kHz were applied, the ion gel dielectric enabled the contact angle reduction to 89.6° (Figure 2). This shows the significantly enhanced angle change Δ*θ* = 26.4° due to its high-capacitance characteristic, more than 2-fold improvement, compared to that of a 50 nm SiO_2_ layer. Another interesting observation is the contact angle almost constantly remained as *θ* ≈ 90.9° on the ion gel layer at the frequency range from 1 to 15 kHz (Figure 2). Such AC electrowetting responses result from the unique feature of almost frequency-independent capacitance for the ion gel at the given frequency range [19,36,37]. Consequently, the contact angles correspondingly show an almost negligible variation, although f was largely varied from 1 to 15 kHz. However, this nominal variation in the angle change has not been observed for SiO_2_. At *f* = 15 kHz, the contact angles present almost close to the initial state of *θ*_0_ (i.e., Δ*θ* ≈ 0°) for all thicknesses of the SiO_2_ layer, while the gel layer is still able to achieve the contact angle reduction as large as Δ*θ* = 23.8° (Figure 2). Above 15 kHz, the gel layer dominantly works in a resistive manner due to limited response time in polarization and thus its specific capacitance value sharply drops with f, correspondingly approaching *θ*_0_ at 100 kHz (Figure 2). These experimental results in high-frequency AC electrowetting were further compared to the DC case. Since an applied DC bias voltage fully contributes to the angle change, much larger angle modification can be achieved for the DC case than all AC cases. For example, the gel layer allowed the angle change as large as Δ*θ* = 32.8° at the DC case (Figure 2), while just Δ*θ* = 26.4° at 1 kHz (Figure 2). However, even this angle reduction performance at 1 kHz on the gel layer is still larger than the one (Δ*θ* = 20.8°) under the DC case for a 50 nm SiO_2_ layer.

**Figure 3:**
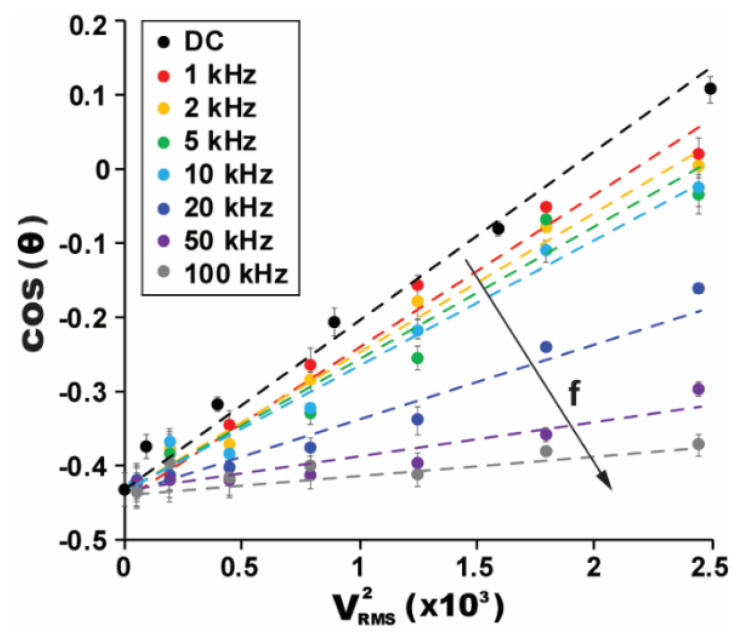
High-frequency AC electrowetting performance varied with AC frequency (*f*) and voltage (*V*_RMS_). A device was fabricated with an 8.3 μm gel layer covered with a 150 nm Teflon layer. While varying *f* and *V*_RMS_, the droplet’s contact angle was measured. For the AC frequencies from 1 to 10 kHz, the variations in the slope are not significant. Beyond this frequency, the decreasing trend in the slop is slow.

Our next study was conducted to see the dependency of the AC frequency (*f*) and root-mean-square voltage (*V*_RMS_) on contact angle modification. Figure 3 presents the measured contact angles in terms of cos(*θ*) varied with the square of the AC voltage input (i.e., *V*_RMS_^2^). Since an initial contact angle is larger than 90° on a hydrophobic surface, the negative value of cos(*θ*) is shown at *V*_RMS_^2^ = 0. As increasing *V*_RMS_, larger electric energy is stored across the ion gel dielectric layer, resulting in the contact angle decreased and the value of cos(*θ*) increased. One interesting observation is the linear relationship between cos(*θ*) and *V*_RMS_^2^. The slope in such a graph of cos(*θ*) versus *V*_RMS_^2^ represents the capacitance effect of the gel layer used, as indicated by the Young-Lippmann Equation (1). The sharpest slope is presented for the DC case. Then, the slope becomes shallower as increasing *f* from 1 to 100 kHz. This decreasing trend in the slope results from the capacitance effect of the gel layer with *f* (i.e., poor contact angle modulation). Another feature attracting our interest is the slopes very close at the frequency range from 1 to 10 kHz, indicating that the gel layer capacitance is almost frequency independent. This frequency-independent characteristic of the ion gel for the capacitance is also supported by the experimental results in Figure 2, where the contact angles on the gel layer have a nominal variation at the frequency range from 1 to 15 kHz. Above 20 kHz, a resistive response of the ion gel dominates, and the slopes of cos(*θ*) drastically decrease with *V*_RMS_^2^. These high-frequency AC electrowetting performances result from the unique characteristics for the ion gel’s capacitance, which features very differently from a conventional dielectric of SiO_2_.

**Figure 4:**
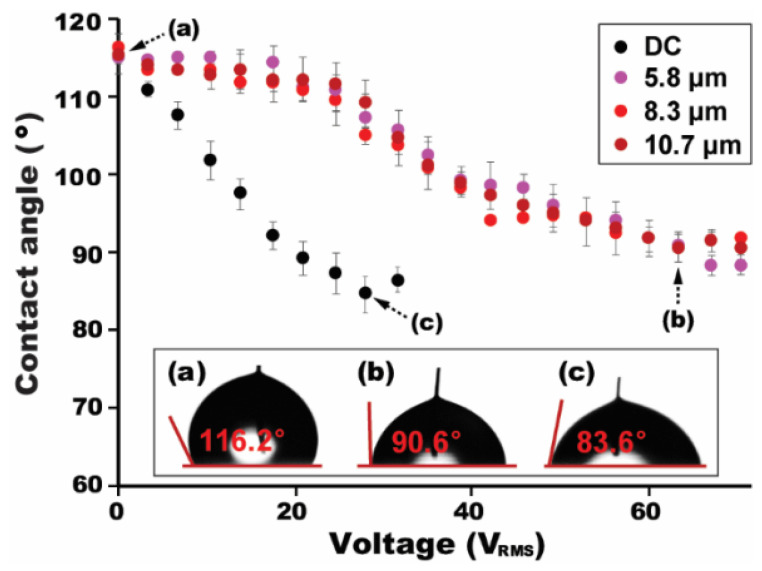
Thickness-independent contact angle modulation. Although the gel layer thickness was largely varied from 5.8 to 10.7 μm, the variation in contact angles is nominal for different layer thicknesses. For the DC situation, such a decreasing trend in the contact angles is much steeper. An applied AC frequency was fixed at 5 kHz for all experiments.

We have further studied the thickness effect of the gel layer on high-frequency AC electrowetting, while varying *V*_RMS_ at a fixed *f* = 5 kHz. Figure 4 shows an initial contact angle of the droplet without the voltage application. With the increase in a bias voltage, the contact angle correspondingly decreases. As described previously, the capacitance of the gel layer is independent on the layer thickness because it is solely contributed by the thickness of the EDLC, not determined by the thickness of the gel layer itself [19]. Consequently, an almost negligible variation in the contact angles is observed in Figure 4, although the gel layer thickness was largely varied from 5.8 to 10.7 μm. These experimental results were further compared to the ones in the DC case. A decreasing trend in the contact angle is much steeper for the DC situation than the AC. For example, the saturation voltage is observed at *V*_sat_ = 63.6 V for the AC (Figure 4), while at *V*_sat_ = 28.3 V for the DC (Figure 4) much earlier than the AC case. In addition, it is worth to note that we have not observed any bubbles caused by electrolysis for both DC and AC cases by using the ion gel as a dielectric layer at a given range of *V*_RMS_.

### 4.2. Low-Frequency AC Electrowetting with the Ion Gel

**Figure 5:**
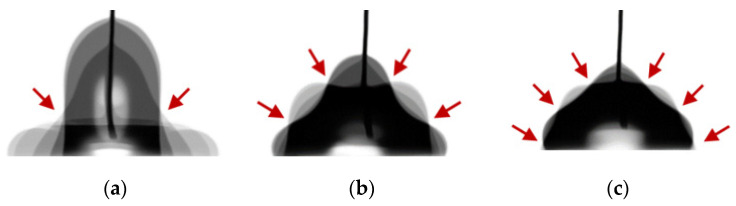
Frequency-dependent oscillation patterns of a water drop. Low-frequency AC electrowetting performance was studied with a 15 μL water drop placed on an ITO-coated glass substrate covered with 8.3 μm ion gel and 150 nm hydrophobic layers, respectively. An AC input voltage keeps constant at *V*_RMS_ = 26.5 V, while the applied AC frequency (*f*) was varied. At a low-frequency range, the droplet mainly undergoes its oscillation whose shape relies on *f*. The oscillation amplitude is noticeably enlarged at corresponding resonance frequencies of (**a**) *f*_1_ = 25 Hz, (**b**) *f*_2_ = 75 Hz, and (**c**) *f*_3_ = 120 Hz, and shows a decreasing trend with the resonance frequency. The arrows indicate the oscillation node points.

When a low-frequency AC signal (typically less than a few hundred Hz) is applied, a droplet experiences its oscillation on a hydrophobic-coated dielectric layer [24,25,26]. This oscillation phenomenon results from the time-harmonic electric input which alternates the Maxwell stress exerted on the three-phase contact line of the droplet. To understand the capacitance effects of the ion gel on low-frequency AC electrowetting performance, the same device, consisting of an 8.3 μm gel layer covered by a 150 nm hydrophobic layer on an ITO-coated glass substrate, was prepared. Figure 5 presents the droplet’s oscillation patterns relying on *f* (see Video S1) when *V*_RMS_ = 26.5 V. Each image presents a superposition of over 5 images taken by a high-speed camera during a half period of the oscillation. It is observed that the oscillation amplitude is maximized at corresponding resonance frequencies of *f*_1_ = 25 Hz, *f*_2_ = 75 Hz, and *f*_3_ = 120 Hz. Droplet’s oscillation shape is also featured with even node points (which are indicated with the arrows in Figure 5) at each resonance frequency.

**Figure 6:**
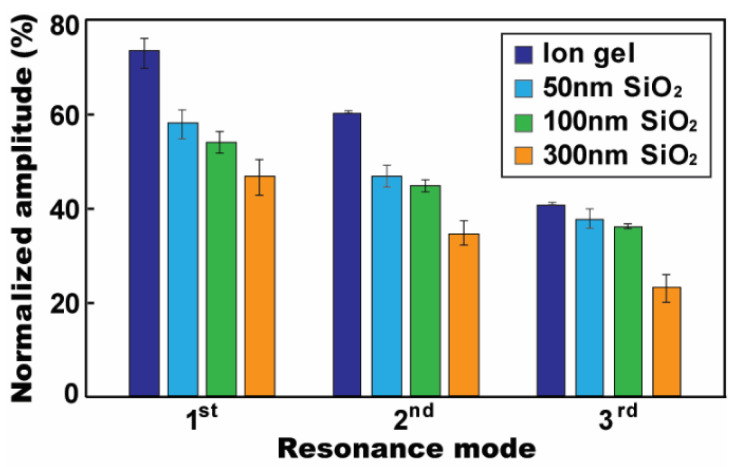
Normalized oscillation amplitude at the first three resonance frequencies of *f*_1_ = 25 Hz, *f*_2_ = 75 Hz, and *f*_3_ = 120 Hz. Oscillation amplitude of the droplet was evaluated on various dielectric layers such as 8.3 μm ion gel, and 50, 100 and 300 nm SiO_2_, while covering with a 150 nm Teflon layer. The use of the ion gel dielectric allows large electric energy stored across its dielectric capacitor, thus enhancing the oscillation performance much larger than a conventional dielectric material of the SiO_2_. A thinner SiO_2_ layer also results in higher oscillation amplitude for all resonance frequencies. All experiments were conducted at *V*_RMS_ = 26.5 V.

To quantify the low-frequency AC electrowetting performance with the ion gel, the oscillation amplitude of the droplet was characterized by normalizing the droplet size radially elongated by the Maxwell stress to the original base diameter of 3.25 mm. Figure 6 presents the normalized oscillation magnitude at each resonance mode. On the gel layer, the droplet was radially elongated 73.8% more than that of its original base diameter at the first mode of *f*_1_ = 25 Hz. At the second and third modes (i.e., *f*_2_ = 75 Hz and *f*_3_ = 120 Hz), smaller oscillation amplitudes were observed as 60.2% and 40.6%, respectively. For comparative study, the experiment was repeated for a conventional dielectric of SiO_2_. The layer thickness was varied from 50 to 300 nm to provide several different capacitance values of the SiO_2_ layer. It is worth to note that the resonance frequencies for droplet oscillation are not affected by the capacitance of a dielectric layer used [41]. Thus, the oscillation performance on the SiO_2_ layers was characterized at the same resonance frequencies as the ones of the ion gel. Figure 6 shows comparative experimental results. A high-capacitance characteristic of the ion gel enhances the oscillation performance much larger than the SiO_2_ layers for all three resonance modes. At the first resonance mode, the gel layer enabled 27.0% larger oscillation performance than that of a 50 nm thick layer of SiO_2_. When it is compared to a 300 nm SiO_2_ layer, the oscillation performance was enlarged as much as 57.6%. Such enhancement in the droplet’s oscillation is similarly observed at higher resonance modes by using the ion gel layer.

**Figure 7:**
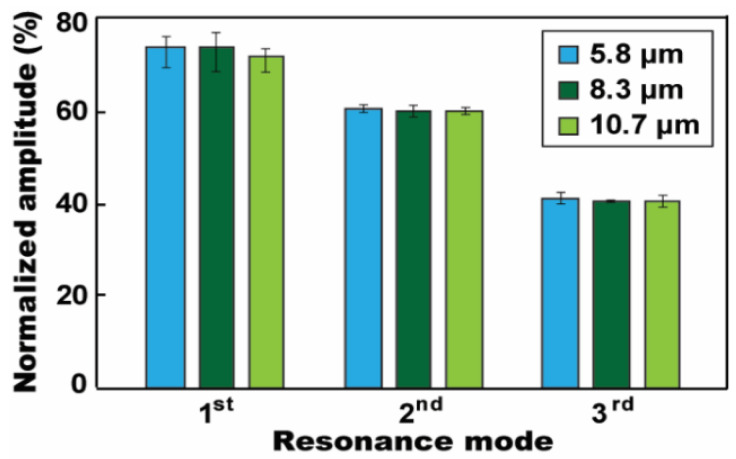
Thickness-independent oscillation amplitude. The droplet’s oscillation performance was evaluated for various thicknesses of the gel layer. An almost negligible variation is observed in the oscillation magnitude of the droplet for all three resonances, although the gel layer’s thickness is largely varied. All experiments were conducted at *V*_RMS_ = 26.5 V.

Figure 6 also presents the thickness-dependent capacitance effect of the SiO_2_ layer on low-frequency AC electrowetting. The oscillation performance increases by using a thinner SiO_2_ layer. These experimental results well follow with our general understanding such that the capacitance of a dielectric layer is inversely proportional to the layer thickness. Thus, a thinner SiO_2_ layer enhances oscillation performance. However, this understanding of thickness-dependent oscillation performance cannot be applied for the ion gel dielectric. Since the capacitance of the gel layer is solely contributed by the nanometer-thick EDLC formed at the electrified interface, it does not rely on its layer thickness, but the thickness of the EDLC [18,19,21]. Due to this unique characteristic of the ion gel dielectric, the low-frequency oscillation performance is independent on the gel layer thickness, which is verified from the experimental results in Figure 7. Although the gel layer’s thickness was largely varied from 5.8 to 10.7 μm, the variation in oscillation magnitude is almost negligible for all three resonance modes.

## 5. Conclusions

AC electrowetting performance dominantly affected by several unique characteristics of the ion gel dielectric for its capacitance was investigated. To understand the effects of the ion gel, experimental studies have been conducted in two separated AC frequency regions. At a high-frequency region above 1 kHz, we have mainly observed the contact angle modification of the droplet. The high-capacitance feature of the ion gel can enhance the contact angle reduction as large as Δ*θ* = 26.4°, showing more than 2-fold improvement compared to that of a 50 nm SiO_2_ layer when *f* = 1 kHz. At the frequency range from 1 to 15 kHz, the droplet’s contact angle keeps almost constantly remained as *θ* ≈ 90.9° on the gel layer. This is because the ion gel dominantly behaves in a capacitive manner up to 15 kHz. As further increasing the AC frequency above 15 kHz, such a capacitive response of the gel layer sharply decreases, resulting in the drastic increase in the contact angle. At a low-frequency region below a few hundred Hz, the droplet mainly undergoes its oscillation because of the time-harmonic AC electric input that alternates the Maxwell stress exerted on the three-phase contact line of the droplet. Oscillation performance was maximized at corresponding resonance frequencies of *f*_1_ = 25 Hz, *f*_2_ = 75 Hz, and *f*_3_ = 120 Hz. Due to the high-capacitance characteristic, the ion gel significantly enlarges the oscillation performance by 73.8% at the 1st resonance mode, which shows 57.6% larger improvement than that of SiO_2_. It was also interestingly observed that the oscillation performance is independent on the gel layer thickness (i.e., capacitance independency), unlike conventional dielectrics. This observation is because the capacitance of the gel layer is solely contributed by the thickness of the EDLC, not determined by the gel layer thickness itself. Our study herein on the unique features of the ion gel dielectric will be potentially helpful for various AC electrowetting applications with the benefits of mixing enhancement, large contact angle modification, and frequency-independent control.

## Supplementary Materials

The following are available online at https://www.mdpi.com/2072-666X/12/3/320/s1, Video S1: Frequency-dependent oscillation patterns of a water drop.
